# Supplementary material for: Explaining the physics of transfer learning in data-driven turbulence modeling
Source: PNAS Nexus. 2023 Jan 23;2(3):pgad015. doi: 10.1093/pnasnexus/pgad015 (PMC9991455; doi:10.1093/pnasnexus/pgad015)
Supplement: pgad015_Supplementary_Data [file pgad015_supplementary_data.pdf]

Supporting information for "Explaining the physics of transfer learning in data-driven turbulence modeling"

Adam Subel[1*], Yifei Guan[1], Ashesh Chattopadhyay[1], and Pedram Hassanzadeh[1,2†]

[1]Department of Mechanical Engineering, Rice University, Houston 77005 TX

[2]Department of Earth, Environmental and Planetary Sciences, Rice University, Houston 77005 TX

*Current affiliation: Courant Institute of Mathematical Sciences, New York University, New York City 10012 NY
†pedram@rice.edu

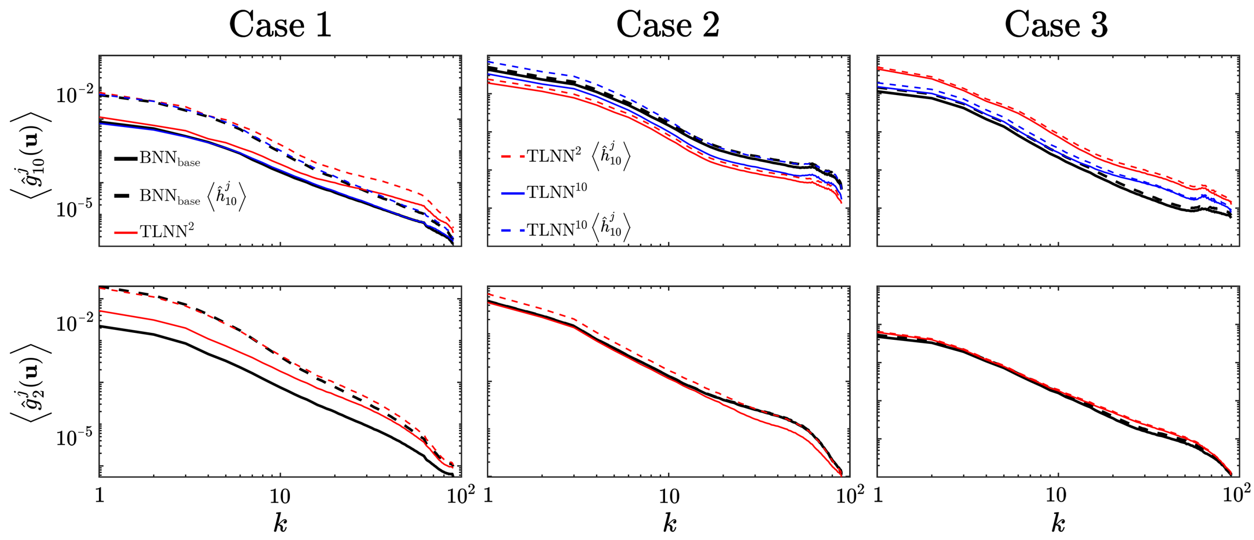

Figure S1: The spectra of all-channels-averaged activations from the first and last hidden layers, $\ell = 2$ and 10, before and after applying the ReLU nonlinear activation function, σ. Before applying the ReLU function, all operations are linear, generating h^j_ℓ (Eq. (7)), whose spectra are shown with dashed lines. The solid lines show the spectra of $g^j_\ell = \sigma(h^j_\ell)$ (Eq. (4)). We emphasize that in this analysis, the same input samples $\mathbf{u}$ from the target flow are used for all 3 networks, thus isolating the effect of changes in weights $\widehat{\hat{W}}^{\beta,j}_\ell$ and biases $\hat{b}^j_\ell$ in Eq. (8). Note that the bottom row only shows activations from TLNN^2 as the activations at layer 2 from the TLNN^{10} are the same as those of the BNN$_{base}$ for $\ell < 10$. In Cases 2 and 3, the ReLU nonlinearity does not alter the activation's spectra significantly, i.e., the change in spectra from TL comes almost entirely from changes to the spectra of the kernels, $\widehat{\hat{W}}^{\beta,j}_\ell$ (changes in $\hat{b}^j_\ell$ are found to be small). In contrast, in Case 1, the ReLU function is an important part of both the BNN$_{base}$ and the TLNNs, resulting in substantial changes in the activations.

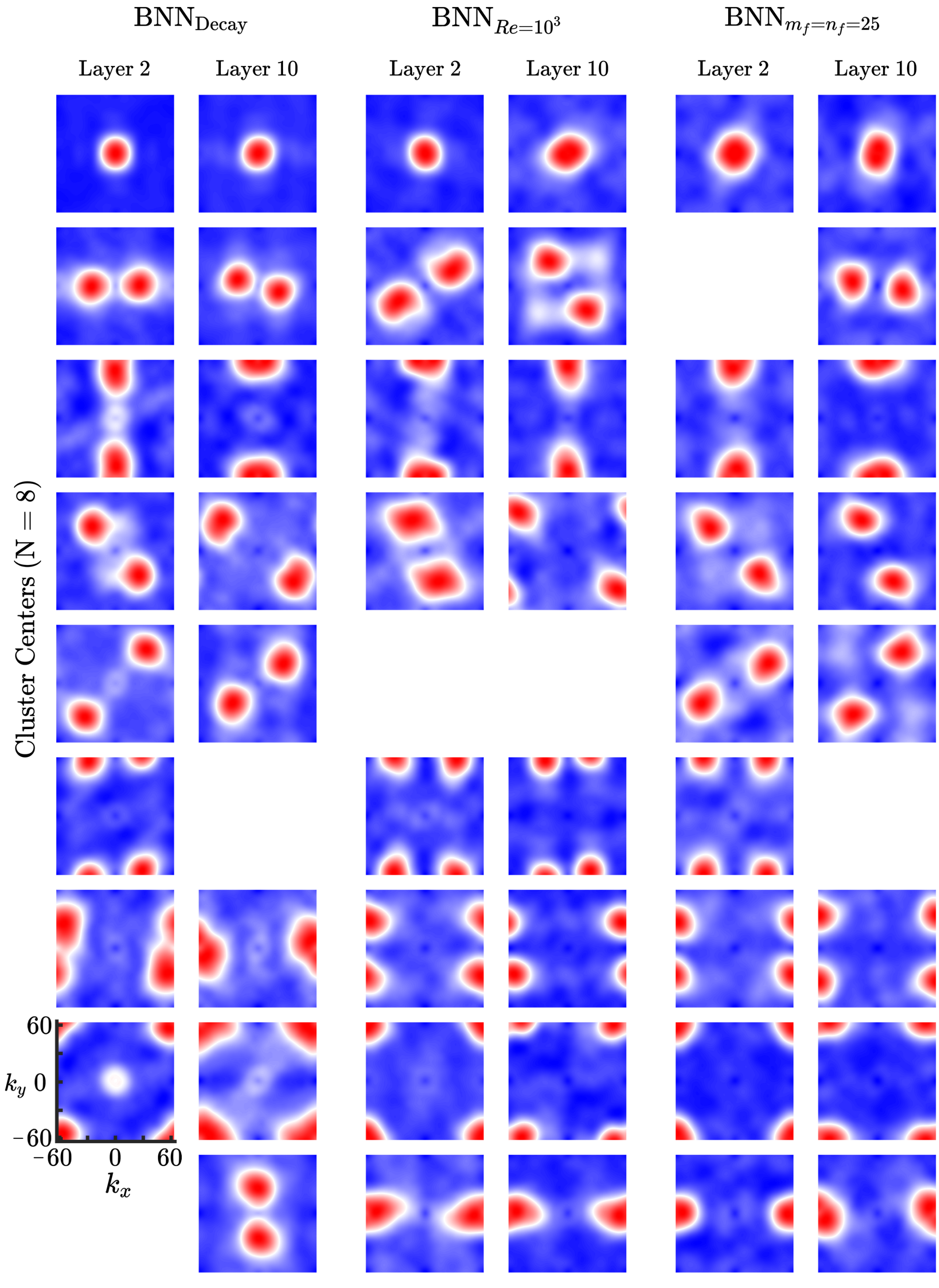

Figure S2: The eight cluster centers of the filters' spectra obtained from applying the k-means algorithm to the 64^2 matrices $\left|\widehat{\widetilde{W}}_{\ell}^{\beta,j}\right|$ of layers 2 and 10 of the BNN$_{base}$. Increasing the number of clusters leads to (qualitatively) more of similar patterns. Note that blue is 0 and red is 0.5 (see Fig. 4 for the colorbar). This analysis clearly shows that the learned convolution filters are a combination of spectral filters: low-pass (first row), high-pass (bottom rows), and band-pass (middle rows) filters. Many cluster centers are qualitatively similar among the 6 BNN$_{base}$ (though their frequencies could be different). The cluster centers of layer 2 of BNN$_{base}$ are used as the reference.

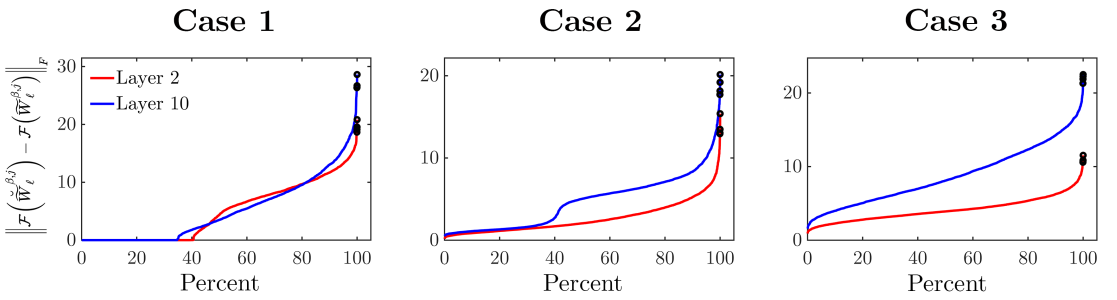

Figure S3: The 3 panels compare the percentile distribution of changes in kernels quantified using the Frobenius norm $\left\|\mathcal{F}\left(\breve{\widetilde{W}}_\ell^{\beta,j}\right)-\mathcal{F}\left(\widetilde{W}_\ell^{\beta,j}\right)\right\|_F$ for a layer. $\mathcal{F}$ indicates Fourier transform (Eq. (5)) and $\breve{\cdot}$ indicates that the weight matrix is from the TLNN (absence of $\breve{\cdot}$ in this figure means that the matrix is from BNN_{base}). In all cases, there is a steep slope, indicating that a few filters change significantly more than the rest (symbols show the 4 most-changed kernel; see Fig. 4.)

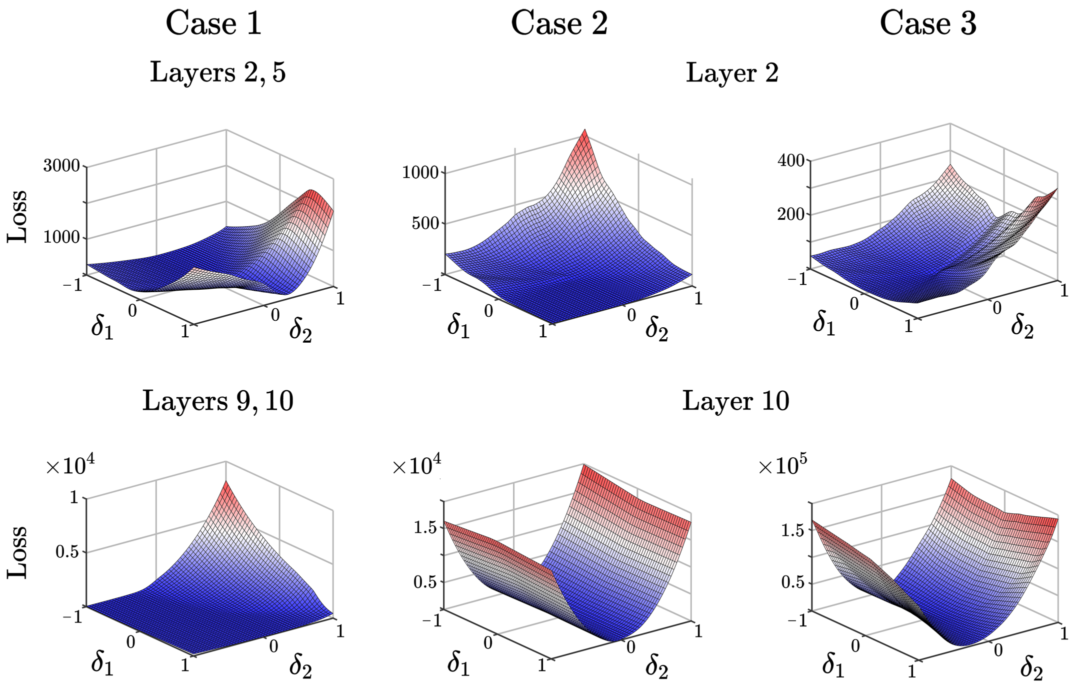

Figure S4: Same as Fig. 5 but using the second method (based on the eigenvectors of the Hessian of $\mathcal{L}$). See Materials and Methods for details. Note large differences in loss magnitudes between the top and bottom rows.

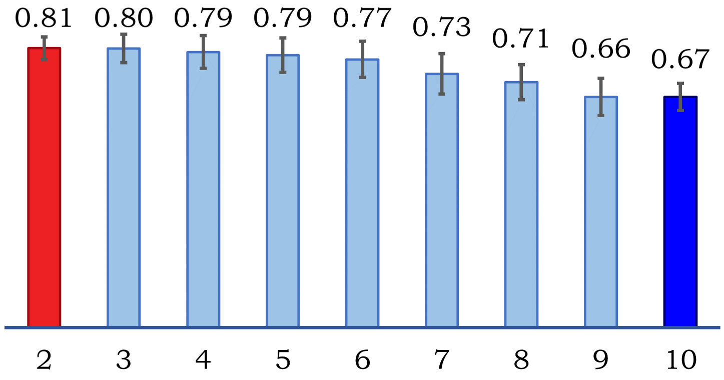

Figure S5: Same as the top-right panel in Fig. 2 except that the base and target systems of Case 3 have been switched. Here, TL is done from a BNN trained on a flow with forcing wavenumber $m_f = n_f = 4$ to a flow with higher forcing wavenumber $m_f = n_f = 25$. The performance follows the same trend as the results in the main text: The shallowest layer, $\ell = 2$ is the best to re-train while the deepest layers, $\ell = 9, 10$, are the worst.
